# Interventions to Minimize Medication Error by Nurses in Intensive Care: A Scoping Review Protocol

**DOI:** 10.3390/nursrep13030091

**Published:** 2023-08-03

**Authors:** Fábio Coelho, Luís Furtado, Natália Mendonça, Hélia Soares, Hugo Duarte, Cristina Costeira, Cátia Santos, Joana Pereira Sousa

**Affiliations:** 1 Department of Nursing, Mental Health, and Gerontology, School of Health, University of the Azores, 9700-042 Angra do Heroísmo, Portugal; luis.cr.furtado@uac.pt (L.F.); helia.m.soares@uac.pt (H.S.); 2 Flores Island Healthcare Unit, 9960-430 Flores Island, Portugal; natalia.lr.mendonca@azores.gov.pt; 3 Center for Innovative Care and Health Technology—ciTechCare, School of Health Sciences, Polytechnic of Leiria, 2411-090 Leiria, Portugal; hugo.s.duarte@ipleiria.pt (H.D.); cristina.costeira@ipleiria.pt (C.C.); catia.santos@ipleiria.pt (C.S.); joana.sousa@ipleiria.pt (J.P.S.)

**Keywords:** interventions, nurses, medications errors, intensive care units

## Abstract

Medication errors represent a concern for healthcare organizations due to their negative consequences. In the nursing context, these errors represent a threat to the quality of care and patient safety. Many factors have been identified as potential causes for these errors in intensive care units. A scoping review will be developed to identify interventions/strategies to minimize the occurrence of medication errors by nurses, considering the Joanna Briggs Institute (JBI) methodology. A search will be conducted in the EbscoHost (CINAHL Complete and MEDLINE), Embase and PubMed databases. Data analysis, extraction and synthesis will be carried out by two reviewers independently. This review will attempt to map which interventions are more specific to minimizing medication error by nurses in intensive care and to recognize which factors influence this type of error to mitigate practices that may lead to error. This protocol acts as the framework for a scoping review in the strategy to map the interventions and which factors contribute to the medication error by intensive care nurses. This study was prospectively registered with the Open Science Framework on 21 April 2023 with registration number DOI 10.17605/OSF.IO/94KH3.

## 1. Introduction

Safety in healthcare settings is a critical area that demands careful consideration and continuous improvement, as implementing a safety culture requires significant changes in attitudes and behaviors [1]. Hospital errors pose a serious threat to patient safety and public health [2]. Patient safety should be regarded as a fundamental skill set that all healthcare professionals must possess, and healthcare organizations must prioritize this aspect [3].

Nursing errors encompass wrong decisions, omissions or actions for which nurses are accountable, resulting in adverse or potentially adverse consequences for the patient during the planning and delivery of care [4,5]. To enhance patient outcomes, recent studies underscore the importance of preventing nursing errors and adverse events. For instance, the implementation of a medication error reduction program in a hospital setting resulted in a noteworthy decrease in medication errors and an improvement in patient safety [6]. Similarly, systematic reviews have shown that interventions aimed at reducing medication errors and adverse drug events in hospitals can significantly enhance patient outcomes and decrease healthcare costs [7].

Preventing nursing errors and adverse events remains a critical aspect of providing safe and effective patient care, making this area a particularly important one. Ongoing research and development of interventions to reduce these events continue to be an important area of focus in the field of nursing. The taxonomy for error reporting, Root Cause Analysis and Analysis of Practice Responsibility (TERCAP), a system that aims to classify nursing errors and the factors that may lead to the error [8], presents nursing errors in eight categories, in which the medication error can be found. Thus, a medication error is an unintentional failure in the medication process, which may arise from the prescription’s dispensing, arming, preparation or administration [9,10], being considered as any avoidable event that may cause harm to the patient through the inappropriate use of medication [9].

High-risk situations are often associated with unsafe practices in the medication management process, leading to medication error, where predisposing factors such as the use of high-risk medications requiring high vigilance, patient factors and environmental factors stand out [11]. Medication errors can lead to suffering, harm (from mild to severe harm) [12,13], disability or even death in 6.5% of hospitalized patients [14].

Medication errors are one of the factors causing the most unintentional harm in hospitalized patients, with around 400,000 medication errors reported annually in the United States of America (USA) [15].

Preventable medication errors associated with injectable medication errors are estimated to increase annual costs in the USA alone by $2.7 billion to $5.1 billion, with an average of $600,000 extra costs for each hospital [16]. Worldwide, the costs that are associated with medication error account for almost 1% of total global health spending, meaning that, globally, it is an estimated cost of around $42 billion [17]. The magnitude of these amounts is overwhelming, especially as it increases the total health expenditure in a resource scarcity, which translates into inefficiency.

In Germany and England, medication error rates of 4.78% and 3.22% were observed, respectively. On the American continent, these rates reached up to 30.4%, with the exception of the USA, which has an average of 5.64%. In Brazil, high rates of 64.3% were recorded, for which medication errors were mostly reported in the preparation and administration of medications [18].

The use of the right five is a strategy considered as a key resource to avoid medication errors. It is true that most health professionals, particularly nurses, know and recognize the ‘right five’ (the right patient, the right drug, the right time, the right dose and the right route) as a safety measure in the medication process, but many errors occur even if professionals check the right five [19].

Safe administration of any medication requires that the right medication is prescribed, then dispensed correctly, in the right form and dosage, prepared correctly and administered at the right times and doses to the right person by the right route [20]. The high complexity of this process and the diversity of health professionals involved mean that there are many opportunities for error.

In hospitals, these errors can be made by medical staff, pharmacists and other pharmacy staff as well as nurses in all clinical settings and various contexts [21].

The cause of the error is multifactorial [22]. The factors that contribute to nursing errors can be related to nurses, patients, health systems or teams [8], specifically those related to fulfilling prescriptions, labelling and packaging as well as the nomenclature of products, the composition, the distribution, the administration, their training, their monitoring and their use [9].

Improving healthcare delivery by mitigating factors that contribute to nursing errors in medication administration is necessary [12]. Standardized procedures, following the five rights, keeping lines of communication open, informing patients about the medications they receive, following strict guidelines, improving labeling, focusing on the work environment, reducing workload, avoiding distractions, creating a cultural space for reporting the error, making the support of managers available for error reporting processes and spreading awareness of the importance of this reporting are necessary [3,4,18,23,24]. Ongoing research and development of interventions to reduce these events will continue to be an important area of focus in the field of nursing [4].

Based on the existing literature, it is clear that medication errors are a significant concern in Intensive Care Units (ICUs), primarily due to the complex care processes, healthcare technologies, complex patient needs and responses to therapeutic interventions. Patients in ICUs are often receiving intravenous medications, requiring careful dosage management and calculation, which can lead to medication errors [25].

In ICUs, due to the complexity of care, patients receive intravenous medications, requiring calculation and dosage management [6,26,27]. In this context, those patients, for the most part, are unconscious and, therefore, are unable to report adverse drug reactions [27]. Due to their vulnerability, they are exposed to medication errors related to working conditions, organizational climate and physical aspects of the work environment, such as poor lighting, poor thermal and acoustic aspects [27] and a high nurse-to-patient ratio [27,28]. Moreover, inadequate supervision [29], pressure, organizational problems, fatigue, lack of time [30], knowledge and performance [24] are factors that may be involved in the occurrence of adverse events in ICUs.

Nurses play a crucial role in preventing medication errors, and they need access to safety mechanisms to minimize the occurrence of medication administration errors [2]. Therefore, the objectives of this scoping review are to identify interventions and strategies that can minimize the occurrence of errors by nurses and identify the factors influencing medication errors by nurses. Additionally, the review aims to examine the consequences of medication errors by nurses in ICU settings. By achieving these objectives, this review will provide valuable insights and inputs to design and implement an effective program focused on preventing medication errors and promoting patient safety in adult intensive care units, supported by existing research and evidence.

## 2. Research Question

Which interventions prevent medication errors by nurses, at any stage of the medication management process, in intensive care units?

### Secondary Questions

Which factors predispose nurses to medication errors in intensive care units?

Which consequences and outcomes result from the occurrence of medication errors by nurses in intensive care units?

## 3. Inclusion Criteria

### 3.1. Participants/Population

This review will consider studies focusing on general care nurses, clinical nurse specialists and advanced practice nurses, regardless of the length of professional practice or level of training. Studies whose participants are physicians, pharmacists, nursing assistants or nursing students will not be included.

In the case of studies that include different professional studies, data on nurses will be extracted, where clear in the original document. If this information is not clear or clarified by the author, the study will be excluded.

### 3.2. Concept

The concept of intervention has been little studied, and many take it for granted in different areas, making little contribution to its definition [31]. An intervention represents a specific set of activities to put into practice an activity of known dimensions [32]. When an intervention is evaluated as producing the expected results, it can be considered effective for the target population and context [33].

Medication error is defined as a failure in the treatment process and an inappropriate use of medication that can or has the potential to harm or cause patient harm [34]. Harm is defined as temporary or permanent compromising of the physical, emotional or psychological function or structure of the body and/or pain resulting from the need for intervention [35].

This review will consider studies that refer to interventions promoted within the scope of preventing medication errors by nurses. Any intervention or strategy to prevent medication errors resulting from nurses’ actions will not be excluded from the review.

### 3.3. Context

This scoping review will consider studies conducted in adult intensive care units. Studies referring to intermediate care units or pediatric and neonatal intensive care units will not be included. Studies conducted in adult ICUs directly involved in the COVID-19 response will be excluded because the risk of medication errors may be higher during times of pandemic due to system failures, inefficient preparedness plans, staff stress and attrition as well as new clinical complexities, requiring differentiated mitigation responses [36].

### 3.4. Types of Studies

This review will include primary, quantitative, qualitative and mixed methods peer-reviewed studies that meet the inclusion criteria. The review will also include review, quantitative, qualitative or mixed methods studies, provided they are eligible as described above.

Reports or technical documents may also be included if qualified structures, such as government bodies, professional organizations or scientific societies, issue them. Literature reviews, opinion texts, letters to the editor and editorials will be excluded.

## 4. Methods

This review will follow the Joanna Briggs Institute (JBI) methodology for scoping reviews [37,38]. The JBI provides guidelines and suggestions for the conduct of a scoping review, revealing the need to prepare a review protocol, which should precede the scoping review [38,39]. The protocol defines the objectives and methods of the systematic review, enabling the transparency of the process, which restricts the presence of bias in the review and should be prepared separately from the scoping review [38,40]. The results of the article selection process will be reported according to Preferred Reporting Items for Systematic Reviews for the Scoping Reviews (PRISMA-ScR) [41]. The PCC mnemonic (P—“population”, C—“concept” and C—“context”) was used to construct the review question and used to organize the eligibility criteria of the documents to be considered. This review protocol is registered in the Open Science Framework [42].

Any changes to the protocol defined here will be reported and justified in the review report.

### 4.1. Search Strategy

The search strategy described here will seek to locate primary published studies, systematic literature reviews, technical documents or reports from government bodies, professional organizations, scientific societies and other organizations with competence and authority in the matter, published between January 2012 and April 2023. The choice of limiting the search in time is due, on the one hand, to restrictions inherent to the size and availability of reviewers and, on the other hand, to the fact that this period includes most of the publications on the subject; even so, it is understood that this time limit will not harm the quality of the data or the conclusions of the review, since it will be representative of the evidence produced over more than a decade. Without prejudice to the above, we recognize that this limitation of the time period for the inclusion of articles may constitute a limitation of this study, as it does not consider all the evidence published on the subject.

Initially, in order to identify the existence of literature reviews on the same topic, a literature search was performed, but no literature reviews were found that analyze the available evidence on interventions or factors promoting safety conduct in procedures related to the use of medication by nurses. A search on Open Science Framework (OSF^®^, Center for Open Science, Charlottesville, VA, USA) was performed using the words “nursing” AND “medication errors” AND “intensive care unit” AND review. Thus, the feasibility and relevance of conducting this literature review is verified.

Next, a preliminary search, distinct from that conducted to determine the feasibility and relevance of this literature review, limited to the PubMed electronic database, was also conducted to identify relevant articles, which were subjected to text analysis, words in the titles and abstracts as well as index terms and keywords used in their description. The terms extracted from natural vocabulary and descriptors were used in constructing a search strategy and in the PubMed database (Table 1). In the next phase, the search strategy will be extended to include the Cumulative Index to Nursing and Allied Health Literature (CINAHL) Complete (via EBSCO), Medical Literature Analysis and Retrieval System Online (MEDLINE) (via EBSCO) and Excerpta Medica Database (Embase) databases, in addition to the PubMed database. A search of the Open Access Scientific Repositories of Portugal (RCAAP) and Dart-Europe will also be conducted to search for unpublished studies, as well as an online search on websites of professional associations, government bodies and scientific societies, among others, to locate relevant documents to support the review. Finally, references from all documents included in the literature review will be checked to identify any articles that may meet the inclusion criteria. Only studies and documents in English or Portuguese will be included.

### 4.2. Study Selection

The records obtained will be exported to the EndNote 20 reference manager (Clarivate Analytics, Philadelphia, PA, USA), where they will be organized and duplicates removed. Subsequently, the results will be exported to the Rayyan platform (Qatar Computing Research Institute, Doha, Qatar), where duplicates will be checked and removed, following screening and selection of all records. Both programs’ duplicates will be carefully removed after confirmation of real duplication.

All records will be screened by title and abstract, by two reviewers and in a blinded manner, through the available functionality in Rayyan platform; in other words, a single reviewer will not be able to know another reviewer’s decision. Identified records will be reviewed in full by two reviewers and blinded. Conflicts between the reviewers will be resolved through discussion or, in the absence of consensus, by a third reviewer.

A random sample of 25 documents will be obtained, which will be subject to a preliminary analysis for the whole team of reviewers, applying the defined eligibility criteria, followed by a meeting for discrepancy analysis and clarification of these same criteria; this strategy may be repeated until a minimum compliance rate of 75% is obtained [38,39].

The survey results will be reported using the flow diagram established by PRISMA-ScR [41]. The assessment of the methodological quality of the included sources of evidence will not be carried out because it is not foreseen in the JBI methodology for scoping reviews [38].

### 4.3. Data Extraction

Data will be extracted from the documents with a final decision to be included in the scoping review by two reviewers, autonomously, using instruments specifically designed for this review.

The data to be extracted will include a summary description of the documents’ characteristics, in addition to the elements that qualify and support the concept of interest and the answer to the established research questions. A third reviewer will aggregate each pair of extractions on the same document (Appendix A). Description of documents will includes authors, year of publication, location where research was conducted or published, objectives and population or sample size. Regarding the results extracted from the source of evidence, the data extraction instrument will include the type of medication error and respective mitigation interventions, predisposing factors for the error, consequences of the error and other aspects not considered in the previous topics. This instrument may change and adapt due to the dynamic process underlying a literature review if elements and specificities not initially contemplated in the review protocol are detected.

To optimize the data extraction work and the familiarization with the extraction tool, a random sample of three articles will be obtained, subject to preliminary extraction by all authors, followed by an analysis and work meeting to standardize the criteria, namely in the sense of ensuring that reviewers extract from the retained documents the data that are actually relevant to the review, i.e., that answer the research question, meaning that extracted data from the different reviewers involved in the process should match.

Any reviewers’ disagreements will be resolved through discussion or, if the disagreement is not resolved, by a third reviewer.

The results will be compared and discussed to ensure that the data relevant to the review have been effectively extracted and that there is consistency in the analysis among reviewers.

The studies’ authors may be contacted to provide additional information whenever justified.

### 4.4. Data Analysis and Presentation

The results of the articles included in this scoping review will be presented using tables and diagrams supported by narrative descriptions. The extracted data may also be organized in a categorization structure to be defined according to the evolution of the extraction and analysis process of the extracted data.

A description of how the data obtained relates to the review’s objectives will be carried out. A discussion will be held on the review results, regarding the interventions found to minimize medication errors by nurses, as well as the factors that influence them. Conclusions will be drawn from the evidence identified.

## 5. Discussion

Medication errors are a significant global concern that affects patients in various healthcare settings. They can result from various factors, including human error, communication breakdowns, insufficient training, fatigue, distractions and workload pressure. Nursing, in particular, plays a crucial role in medication administration, encompassing approximately 40% of hospital practices [2]. The gravity of the situation becomes apparent when considering the statistics in the USA, where medication errors are responsible for at least one death per day and cause harm to approximately 1.3 million people annually [3]. These errors can occur in any hospital setting and have far-reaching consequences for patients and their families, leading to increased rates of morbidity and mortality [4]. The economic ramifications of medication errors are also substantial [4,43], where medication errors impose a significant financial burden on healthcare systems. The cost associated with adverse events, prolonged hospital stays, and legal liabilities further emphasizes the importance of effective interventions to prevent these errors [16].

The significance of this topic calls for a thorough exploration of the existing body of literature. By conducting a comprehensive literature review, a deeper understanding of the problem and its real-world implications can be gained. Furthermore, such a review aims to identify and analyze a wide range of interventions that have been studied in the past. The insights gained from these interventions will inform the development of a carefully tailored and evidence-based program aimed at preventing medication errors among nurses working in intensive care settings. This undertaking is of utmost importance in enhancing patient safety and optimizing healthcare outcomes in critical care environments.

While acknowledging the importance of all professionals involved in the medication management process, it is crucial to recognize that nurses are at the final stage of this chain. As they are responsible for the preparation and administration of medications, nurses have a fundamental role in preventing errors from reaching the patient [18,21]. Hence, a thorough investigation of nursing-related factors and interventions becomes paramount in addressing medication errors effectively.

The choice to conduct a scoping review in this context is justified by its ability to explore the literature extensively and provide evidence summaries, making it an appropriate method for mapping the evidence, which may be extensive in this complex topic [44,45]. By using this approach, the review aims to comprehensively characterize the factors contributing to medication errors among nurses and identify a diverse range of interventions used to address this issue.

The decision not to assess the methodological quality of the included studies is deliberate, aligning with the main objective of the scoping review. The primary purpose of this review is to describe and map the literature concerning the characteristics and factors related to medication errors among nurses in intensive care settings [38]. Evaluating the methodological quality and excluding studies based on this evaluation could inadvertently lead to the omission of relevant research. Some studies, despite lacking clarity in certain methodological aspects, may still offer valuable insights. Therefore, it is essential to approach the assessment of methodological quality with caution, ensuring a thorough consideration of each study’s potential contributions to the research area. The decision not to assess methodological quality aligns with the goal of conducting a comprehensive review that includes a wide range of relevant studies to address the research question and achieve the overall objective of the review study.

## 6. Conclusions

The scoping review resulting from this protocol is anticipated to make a significant contribution to identifying the underlying factors that compromise safety and lead to medication errors, which can potentially cause harm and negative consequences for patients. Medication errors are a pressing global concern, and addressing them is crucial to improving patient safety and healthcare outcomes. Nurses play a critical role in the medication administration process and are pivotal in preventing errors that could harm patients. By thoroughly examining the literature, this review aims to shed light on the complex web of variables that may influence medication errors in the context of nursing practice within adult intensive care units.

The ultimate goal of this review is to gather relevant and evidence-based information that will support the development of a targeted intervention or strategy to prevent medication errors committed by nurses in intensive care settings. By approaching the literature review with a careful and inclusive mindset, valuable insights can be gained, leading to significant advancements in medication safety and nursing practices. By leveraging the insights gained from the literature, healthcare organizations and policymakers can design and implement proactive measures to enhance patient safety and ensure the delivery of exceptional care in critical care environments.

Recognizing the systematic and dynamic nature of the review process, the research team is committed to transparency and rigor. Any deviations or alterations to the initial scope outlined in this study will be meticulously documented and justified in the final literature review report. This report, which will be published upon the completion of the review, serves as a testament to the integrity of the methodology employed throughout the study.

The comprehensive literature review report will not only summarize the findings but also provide readers with valuable insights into the evolution of the review process. By documenting the rationale behind any decisions that may have departed from the original terms, readers will gain a deeper understanding of the complexities and nuances involved in conducting such a review. This commitment to transparency ensures that the research process is robust and accountable, ultimately enhancing the credibility and reliability of the review’s findings.

Moreover, this review report can serve as a valuable resource for future research endeavors, providing a comprehensive overview of the current state of knowledge on medication errors in intensive care settings. Researchers, practitioners and policymakers can draw upon the report’s insights and recommendations to inform their own initiatives aimed at improving patient safety and healthcare outcomes.

By publishing the comprehensive literature review report, the research team aims to contribute to the broader field of patient safety and medication management in healthcare. Ultimately, the dissemination of this research serves to promote the culture of safety, excellence in care and continuous improvement, thus advancing the overall quality of healthcare delivery and patient outcomes.

## Figures and Tables

**Table 1:** Search Strategy for PubMed.

Search	Query	Results
#9	#3 AND #4 AND #7 from 2012–2023	202
#8	#3 AND #4 AND #7	370
#7	#5 NOT #6	223,706
#6	“intensive care units, pediatric” [Mesh] OR “intensive care units, neonatal” [Mesh] OR “intensive care units, pediatric” [TIAB] OR “intensive care units, neonatal” [TIAB]	27,043
#5	“intensive care units” [Mesh] OR “respiratory care units” [Mesh] OR “coronary care units” [Mesh] OR “intensive medical care *” [TIAB] OR “intensive care *” [TIAB] OR ICU [TIAB] OR “care, intensive” [TIAB] OR “intensive care unit *” [TIAB] OR “intensive care medicine” [TIAB] OR “respiratory care unit *” [TIAB] OR “coronary care unit *” [TIAB]	250,749
#4	“medical errors” [Mesh] OR “medication errors” [Mesh] OR “nursing error *” [TIAB] OR “medical error *” [TIAB] OR “medication error *” [TIAB] OR “medication administration error *” [TIAB] OR “medication preparation error *” [TIAB]	126,240
#3	#1 NOT #2	463,685
#2	Physicians [Mesh] OR students [Mesh] OR “nursing assistants” [Mesh] OR physician * [TIAB] OR student * [TIAB] OR “nursing assistant *” [TIAB] OR “nursing student *” [TIAB] OR “medical student *” [TIAB] OR undergraduate [TIAB] OR “nursing aide *” [TIAB] OR “nursing assistant *” [TIAB]	936,356
#1	“nurses” [Mesh] OR nurs * [TIAB]	559,604

* reports to words search truncation in PUBMED.

## Data Availability

Not applicable.

## Public Involvement Statement

No public involvement in any aspect of this research.

## Guidelines and Standards Statement

This manuscript was drafted against the PRISMA-ScR (Tricco AC, Lillie E, Zarin W, O’Brien KK, Colquhoun H, Levac D, Moher D, Peters MDJ, Horsley T, Weeks L, Hempel S, Akl EA, Chang C, McGowan J, Stewart L, Hartling L, Aldcroft A, Wilson MG, Garritty C, Lewin S, Godfrey CM, Macdonald MT, Langlois EV, Soares-Weiser K, Moriarty J, Clifford T, Tunçalp Ö, Straus SE. PRISMA Extension for Scoping Reviews (PRISMA-ScR): Checklist and Explanation. Ann Intern Med. 2018. PMID: 30178033) for scoping review research.

## Appendix A. Instrument for Extracting the Characteristics of the Study

**Scoping Review Details**	
Scoping review title	Interventions to minimize medication error by Nurses in intensive care: a scoping review protocol
Review objective(s)	(a) identify interventions/strategies to minimize the occurrence of errors by nurses and facilitators of a safety-promoting intervention. (b) map the factors influencing medication error by nurses. (c) identify the consequences of the medication error by nurses, in an intensive care unit context
Review question(s)	Which interventions prevent medication errors by nurses, at any stage of the medication management process, in intensive care units? Which factors predispose nurses to medication errors among in intensive care units? Which consequences and outcomes result from the occurrence of medication errors by nurses in intensive care units?
**Inclusion/exclusion criteria**	
Population	This review will consider studies that include nurses (general care, specialist/parental, advanced practice nurses) regardless of the length of professional practice or level of training.
Context	This review will consider studies conducted in adult intensive care units (multipurpose intensive care, coronary intensive care, neurocritical intensive care and respiratory intensive care).
Concept	This review will consider studies that refer to interventions promoted within the scope of preventing medication errors by nurses.
Types of evidence source	This scoping review will consider any quantitative, qualitative and mixed methods study designs for inclusion. Moreover, systematic reviews will be considered for inclusion in the proposed scoping review.
**Evidence source details and characteristics**	
Author(s)	
Year of publication	
Origin/country of origin (where the source was published or conducted)	
Aims/purpose	
Population and sample size	
**Details/Results extracted from the source of evidence** (concerning the concept of the scoping review)	
Interventions/strategies used in the prevention of medication error by nurses	
Type of Error (E)	Mitigation strategies
E1	Mitigation strategies for E1
E2	Mitigation strategies for E2
E3	Mitigation strategies for E3
(…)	(…)
Predisposing factors for the error	
Consequences/outcomes of the error	
Other aspects (not considered above)	
